# Cocaine Enhances DC to T-cell HIV-1 Transmission by Activating DC-SIGN/LARG/LSP1 Complex and Facilitating Infectious Synapse Formation

**DOI:** 10.1038/srep40648

**Published:** 2017-01-17

**Authors:** Anil Prasad, Rutuja Kulkarni, Shuxian Jiang, Jerome E. Groopman

**Affiliations:** 1 Division of Experimental Medicine, Beth Israel Deaconess Medical Center, Harvard Medical School, Boston, MA 02215, USA

## Abstract

DC-SIGN is a dendritic cell surface structure which participates in binding and transmission of HIV-1. Here, for the first time we demonstrate that cocaine induces over expression of DC-SIGN and significantly enhances virus transfer from DCs to T-cells by increasing the binding and internalization of HIV-1 in DCs. We found that cocaine activates a DC-SIGN mediated ‘signalosome’ complex by enhancing its association with LARG and LSP1. Further, LARG was observed to participate in DC-SIGN mediated internalization of HIV-1 in DCs. Intracellular trafficking studies of HIV-1 in cocaine treated DCs revealed increased co-localization of HIV-1 with endosomal or multi vesicular body (MVB) markers such as CD81 and VPS4 and decreased co-localization with the phagolysomal marker LAMP1; this signified altered intracellular trafficking and decreased degradation of HIV-1 in cocaine treated DCs. Furthermore, we found that cocaine induced activation of LARG which in turn activated Rho A and the focal adhesion molecules FAK, Pyk2 and paxillin. This signaling cascade enhanced the formation of an infectious synapse between DCs and T-cells. Our study provides insight into the molecular mechanisms of cocaine’s contribution to key components in HIV pathogenesis and highlights novel targets for interrupting the virus life cycle in substance using hosts.

Substance abuse poses a major challenge for the eradication of the HIV/AIDS pandemic^1^,^2^,^3^,^4^,^5^,^6^,^7^. Cocaine is a commonly used illicit drug and prominently linked to HIV-1 infection and spread by both fostering high risk behaviors and facilitating the pathobiology of the virus^1^,^2^,^3^,^4^,^5^,^6^,^7^. Prior studies have shown that cocaine enhances viral replication in various cell types and alters the immune response by regulating the secretion of cytokines and expression of their receptors, accelerating the decline of CD4+ T-cells and disrupting the integrity of the blood-brain barrier^4^,^8^,^9^,^10^,^11^,^12^,^13^,^14^,^15^,^16^,^17^,^18^. However, the molecular mechanisms whereby cocaine may act as a cofactor for HIV-1 pathogenesis are not fully defined.

*In vivo* studies in a humanized mouse model revealed that cocaine significantly enhanced HIV-1 infection and increased the circulating viral load^17^,^19^. Several *in vitro* studies have demonstrated enhanced HIV-1 infection and replication in T-cells and monocyte-macrophages in the presence of cocaine^13^,^14^,^15^,^20^,^21^. The drug is also known to regulate cytokine secretion and function by suppressing the secretion of chemokines such as RANTES, MIP-1a and MIP-1b, which can inhibit HIV-1 infection in target cells^11^,^12^. Increased expression of HIV-1 co-receptors CXCR4 and CCR5 has been observed in cocaine treated cells, which may facilitate viral entry into the target cells^11^,^12^. Recent studies on cellular miRNA species in cocaine treated cells have revealed that cocaine down regulated miR-125b, known to inhibit viral replication in CD4+ T-cells by blocking translation of viral specific proteins^10^,^22^. Decreased expression of miR-155 has also been observed in cocaine treated monocyte-derived dendritic cells, thereby altering expression of DC-SIGN^13^. Moreover, cocaine-using HIV-1 infected patients exhibit significantly higher levels of DC-SIGN in dendritic cells compared with cocaine non-using HIV positive patients^23^.

DC-SIGN belongs to C-type lectin groups primarily expressed on dendritic cells and plays an important role in sequestration of HIV-1 virions^24^,^25^,^26^. DC-SIGN captures HIV-1 through a high affinity interaction with HIV-1 gp120 and facilitates its internalization into an intracellular non-lysosomal compartment termed an ‘endosome’ or ‘signalosome’^27^,^28^. Some virions are trafficked into multi-vesicular bodies (MVB) that facilitate in *trans* transmission to CD4+ T-cells^29^,^30^,^31^. Alternatively, endocytic virions can also fuse with a phagolysosomal complex and undergo proteasomal degradation^29^,^32^,^33^. DC-SIGN mediated internalization of HIV-1 also activates the DC-SIGN signaling cascade involving Rho-GTPases which enhance the formation of an infectious synapse^34^. An infectious synapse is a complex contact between DCs and T-cells similar to an immune synapse that forms during MHC class II antigen presentation^35^,^36^,^37^. These specialized synapses facilitate rapid transmission of intracellular pathogens, including HIV-1, and protect it from the host immune system^38^,^39^. An infectious synapse is critical for transmission of HIV from DCs to CD4+ T-cells, even when viral titer is very low^38^,^39^.

Several downstream molecular components are involved in the DC-SIGN mediated internalization of viral particles; stimulation of DC-SIGN by HIV-1 gp120 activates the Rho guanine nucleotide-exchange factor, LARG, which in turn activates Rho-GTPases and recruits scaffold molecules such as Leukocyte specific protein 1(LSP1), KSR1, CNKs and Rho to form a signalosome complex^27^. This complex may be responsible for further intracellular trafficking of endocytic compartments containing virions^27^.

Here we studied molecular mechanisms involved in how cocaine may enhance DC to T-cell HIV-1 transmission and replication in T-cells. We found that cocaine activates DC-SIGN/LARG and alters intracellular trafficking machinery which results in the increased internalization of HIV-1 and rapid transmission of HIV-1 through an infectious synapse.

## Results

### Cocaine enhances the transmission of HIV-1 from DCs to T-cells

**Figure 1:**
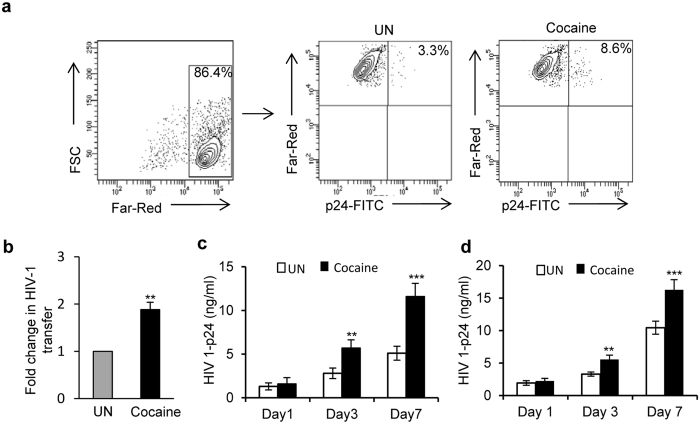
Cocaine enhances the transfer of HIV-1 from DCs to T-cells. (**a**) HIV-1 transmission from DCs to T-cells by flow cytometry. Cocaine (1 μM) pre-treated or untreated DCs were incubated with HIV-1 BaL for 2 hours, then washed to remove free virus. Far-Red-labeled T-cells were added with or without 1 μM of cocaine. After 3 days, cells were harvested and stained with p24-FITC antibody (HIV-1 marker) and analyzed by flow cytometry. Far-Red-labeled T-cells were gated (left panel) and p24 positive Far-Red-labeled T-cells were quantitated. Upper right quadrants of center (untreated-UN) and right panels (cocaine treated) represent the percent of T-cells that have internalized HIV-1. (**b**) Fold change in HIV-1 transfer as described in (**a**) by considering untreated as 1. Data represent the mean ± SEM of 3 independent experiments done in triplicate for untreated T-cells vs. those treated with cocaine (p ≤ 0.01, 2-tailed t-test). (**c**) Quantification of p24 titer in the cell supernatants on day 1, 3 and 5 of DC to T-cell transfer assay as described in (**a**). (**d**) Quantification of p24 titer in the cell supernatants on day 1, 3 and 5 of T-cell to T-cell transfer assay as described in Methods. Data represent the mean ± SEM of 3 experiments done in triplicate for untreated cells vs. cells treated with cocaine (**p ≤ 0.01; ***p ≤ 0.001, 2-tailed t-test).

Cell-to-cell transmission of viral infection is a highly efficient mechanism that can bypass various host resistance factors^39^,^40^. We first tested the effects of cocaine on this process, using an *in vitro* DC to T-cell viral transfer assay. We pretreated the immature monocyte derived dendritic cells (hereafter referred to as “DCs”) with or without 1 μM of cocaine for 2 hours, then incubated with HIV-1 BaL (hereafter referred to as “HIV-1”) for 2 hours, washed the cells to remove unbound virus and added Far Red labelled-T-cells at 1:4 ratio with or without cocaine. After 3 days, the DC and T-cell co-cultures were stained with anti-HIV-1 p24 antibody and analyzed by flow cytometry. We quantitated the HIV-1 p24 positive Far-Red-labelled T-cells in the total Far-Red-labelled T-cell population, which represents T-cells infected from DCs. We found a 2 fold increase in transfer of HIV-1 in the presence of cocaine treated cells compared to untreated cells (Fig. 1a,b, p = 0.009). In addition, we quantified HIV-1 p24 in the cell supernatants on day 1, 3 and 7and found that the titer significantly increased in cocaine treated cells compared to controls (Fig. 1c, p = 0.00091). This result indicates that cocaine significantly enhanced transmission of HIV-1 from DCs to T-cells. Next, we analyzed the effects of cocaine on T-cell to T-cell transfer by using human peripheral blood mononuclear cells derived T-cells, as described in the methods. Though we did not observe any significant change in the percentage of HIV-1 p24 positive Far-Red-labelled T-cells between cocaine treated and untreated cells (data not shown), we observed a significant increase in HIV-1 p24 titer in the cell supernatants treated with cocaine compared to controls (Fig. 1d, p = 0.0001). The effects of cocaine on T-cell to T-cell transfer in Jurkat T-cells were assessed using HIV-1 Gag iGFP virus. In this experiment, we also did not observe any changes in the transfer of virus in presence of cocaine (data not shown). These results indicate that cocaine may exert differential effects on HIV-1 transmission between DC to T-cell and T-cell to T-cell.

### Cocaine enhances binding and internalization of HIV-1 and its effects on host resistance factors in DCs

**Figure 2:**
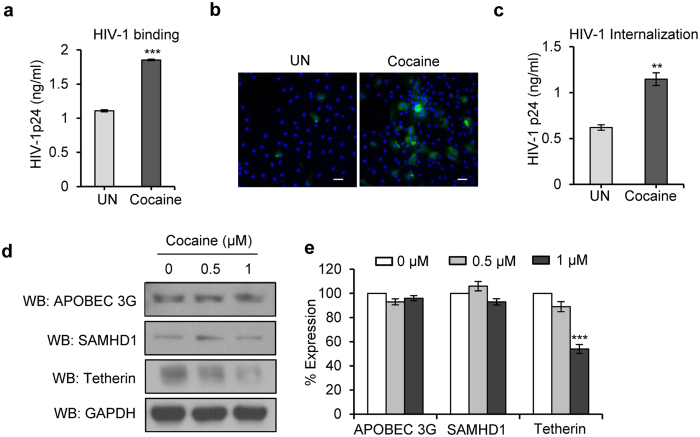
Cocaine increases HIV-1 BaL binding and internalization in DCs. (**a**) For virus binding studies, DCs were untreated or pre-treated with cocaine (1 μM) at 37 °C for 2 hours then incubated with HIV-1 BaL for 2 hours at 4 °C, and the cell lysates were analyzed for HIV-1 p24 levels. Data represent the mean ± SD of 3 independent experiments (***p ≤ 0.001). (**b**,**c**) Cocaine (1 μM) pre-treated or untreated DCs were incubated with HIV-1 BaL for 4 hours at 37 °C. The cells were incubated with trypsin, washed and analyzed under confocal microscopy (Green = HIV-1 p24; Blue = DAPI; Scale bar = 10 μm) or cell lysates were quantitated for p24 titer by using ELISA. Data represent the mean ± SEM of 3 independent experiments for untreated cells vs. cells treated with cocaine (**p ≤ 0.01, 2-tailed t-test). (**d**) Western blot analysis of APOBEC3G, SAMHD1 and tetherin in DCs treated with cocaine (0–1 μM) for 24 hours. GAPDH served as a loading control. (**e**) Quantitative analysis of Western blots of APOBEC3G, SAMHD1 and tetherin in DCs treated with cocaine (0–1 μM) for 24 hours. The band intensity in each lane was determined by densitometry. The fold change was determined by calculating the value of untreated (0) lane as 1. Data represent the mean ± SD of 3 independent experiments (***p ≤ 0.001, 2-tailed t-test).

Since we observed increased HIV-1 transmission from DC to T-cells in the presence of cocaine, we further investigated the mechanisms involved in this enhanced transmission. Both binding and internalization are important steps in the viral life cycle, so we analyzed the effects of cocaine on these processes. We found a significant increase in HIV-1 binding in cocaine treated cells compared to untreated cells (Fig. 2a, p = 0.00038). Similarly, analysis of HIV-1 internalization revealed a more than 2 fold increase in cocaine treated DCs compared to untreated DCs (Fig. 2b,c, p = 0.0011).

Next, we investigated the effects of cocaine on host restriction factors such as APOBEC3G, SAMHD1 and tetherin^32^,^41^,^42^. These are a group of specific cellular antiviral factors that limit replication at various steps of the viral life cycle^32^,^41^,^42^. We treated the DCs at 0–1 μM cocaine for 24 hours and analyzed their expression pattern by Western blotting. There were no significant changes in the expression pattern of APOBEC3G and SAMHD1, however, we found a significant decrease in expression of tetherin in cocaine treated cells (Fig. 2d,e, p = 0.00071). Tetherin is an interferon–inducible cellular factor that inhibits the release of viruses by tethering them on to the cell surface^43^.

### Cocaine induced increased expression of DC-SIGN in DCs and its effects on CXCR4 and CCR5 expression in DCs and T-cells

**Figure 3:**
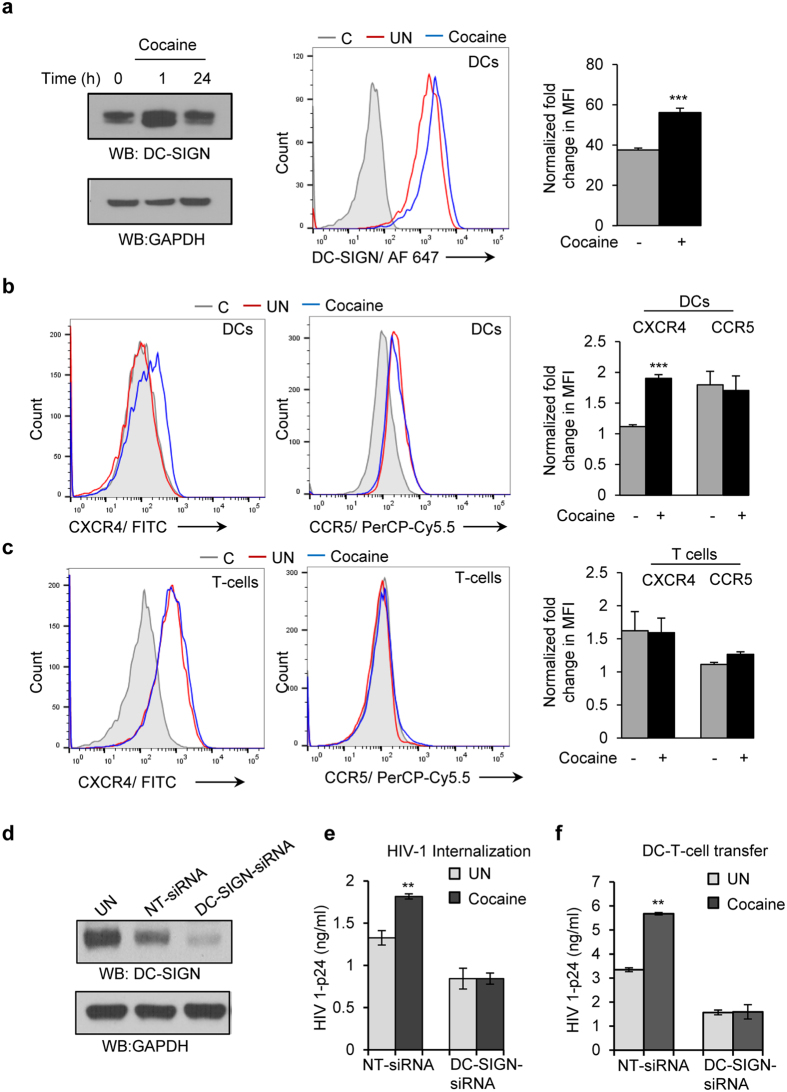
Cocaine-induced increased expression of DC-SIGN in DCs and drug effects on CXCR4 and CCR5 expression in DCs and T-cells. (**a**) Cell lysates from DCs untreated or treated with cocaine (1 μM) for indicated time points were analyzed for DC-SIGN expression by Western blot analysis. GAPDH served as a loading control (**a**, left panel). Cells were analyzed by flow cytometry and representative histogram is shown (**a**, middle panel). Fold change of mean fluorescent intensity (MFI) from 3 independent experiments (**a**, right panel). Fold change in MFI is calculated by normalizing to isotype control (***p ≤ 0.001, 2-tailed t-test). (**b**) DCs were untreated or treated with cocaine (1 μM) for 1 hour and stained with isotype control or CXCR4 (left panel) or CCR5 (middle panel) antibodies, and analyzed by flow cytometry. Fold change of mean fluorescent intensity (MFI) from 3 independent experiments (right panel). Fold change in MFI is calculated by normalizing to isotype control (***p ≤ 0.001, 2-tailed t-test). (**c**) T-cells were untreated or treated with cocaine (1 μM) for 1 hour and stained with isotype control or CXCR4 (left panel) or CCR5 (middle panel) antibodies, and analyzed by flow cytometry. Fold change of mean fluorescent intensity (MFI) from 3 independent experiments (right panel). Fold change in MFI is calculated by normalizing to isotype control. (C- Isotype control; UN- Untreated, Cocaine- Cocaine treated). (**d**) DCs were nucleofected with NT-siRNA (control) or DC-SIGN siRNA and DC-SIGN expression was analyzed by Western blotting. (**e**) HIV-1 internalization assay was performed in above mentioned cells in the presence or absence of cocaine. Cell lysates were quantitated for p24 titer by using ELISA. Data represent the mean ± SEM of 3 experiments for untreated cells vs. cells treated with cocaine (**p ≤ 0.01, 2-tailed t-test). (**f**) HIV-1 transfer from NT-siRNA and DC-SIGN nucleofected DCs to T-cells with or without cocaine. Cell supernatants were quantitated for p24 titer at day 3 by using ELISA. Data represent the mean ± SEM of 3 experiments done in triplicate for untreated cells vs. cells treated with cocaine (**p ≤ 0.01, 2-tailed t-test).

We then examined the effects of cocaine on expression of HIV-1 co-receptors in both DC and T-cells. We treated the DCs with 1 μM of cocaine for 0, 1 and 24 hours and analyzed the expression of dendritic cells specific C type ICAM-3-grabbing non-integrin (DC-SIGN), a main surface receptor in viral binding and internalization^24^,^25^,^26^. Western blot analysis revealed a marked increase in the expression of DC-SIGN at 1 hour upon treatment with cocaine (Fig. 3a, left panel). We further confirmed this result by flow cytometric analysis and found a significant increase in surface expression of DC-SIGN in cocaine treated DCs (Fig. 3a center and left panel, p = 0.00004). Our observation was consistent with a previous report that demonstrated increased expression of DC-SIGN in dendritic cells derived from cocaine using HIV-1 patients^23^. Further, we analyzed the expression of chemokine receptors CCR5 and CXCR4 that can bind HIV-1 in the presence and absence of cocaine by flow cytometry. We did not observe a significant difference in the expression pattern of CCR5 and CXCR4 in T-cells upon cocaine treatment (Fig. 3c); however we observed a moderate increase in expression of CXCR4 and no change in CCR5 expression in DCs treated with cocaine compared to control (Fig. 3b). These results indicate that cocaine may modulate expression of DC-SIGN in DCs. Next, we knocked down expression of DC-SIGN expression by using siRNA in DCs and analyzed the effect of cocaine on HIV-1 internalization and HIV-1 transfer from DC to T-cells. We did not observe any significant change in the HIV-1 internalization (Fig. 3e) or HIV-1 transfer from DC to T-cells (Fig. 3f) in DC-SIGN-siRNA transfected DCs with or without cocaine treatment. However, a significant increase in HIV-1 internalization (Fig. 3e, p = 0.0013) and its transfer to T-cells (Fig. 3f, p = 0.0034) was observed in cocaine treated NT-siRNA (control) transfected DCs compared to untreated NT-siRNA (control) transfected DCs. We confirmed the siRNA mediated knockdown of DC-SIGN in DCs by Western blot analysis (Fig. 3d). These results indicate that cocaine mediated increase in expression of DC-SIGN may be responsible for the increased HIV-1 internalization and its transfer to T-cells in cocaine treated DCs.

### Cocaine enhances HIV-1 internalization via activating DC-SIGN/LARG/LSP1 complex

**Figure 4:**
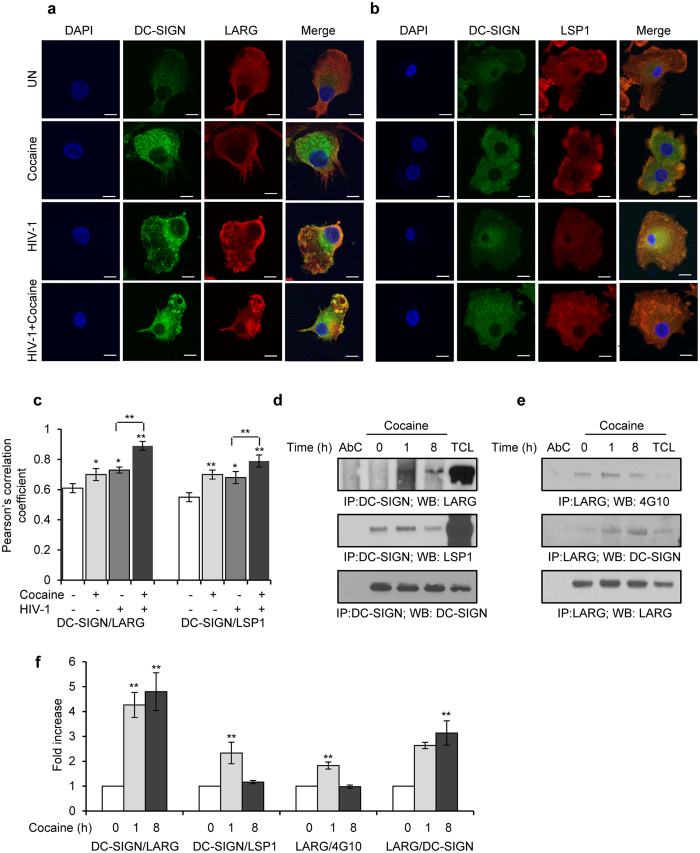
Cocaine enhances formation of DC-SIGN/LARG/LSP1 complex. (**a**) Confocal images of DC-SIGN and LARG interaction in DCs, untreated or treated with cocaine alone, or incubated with HIV-1, or treated with cocaine and incubated with HIV for 24 hours. Scale bars = 10 μm. (**b**) Confocal images of DC-SIGN and LSP1 interaction in DCs, untreated or treated with cocaine alone, or incubated with HIV-1, or treated with cocaine and incubated with HIV for 24 hours. Scale bars = 10 μm. (**c**) Quantitative analysis of the colocalization of DC-SIGN with LARG and DC-SIGN with LSP1 in DCs, under conditions identical to (**a**,**b**), using ImageJ2 software. Data represents mean of Pearson’s correlation coefficient indices of 10 randomly chosen images per condition (*p ≤ 0.05, **p ≤ 0.01, ***p ≤ 0.001, 2-tailed t-test). (**d**) DCs were untreated (0) or treated with cocaine (1 μM) for indicated time points and cell lysates were immunoprecipitated with DC-SIGN antibody and subjected to Western blot analysis using LARG and LSP1 antibodies. DC-SIGN served as a loading control. (**e**) DCs were untreated (0) or treated with cocaine (1 μM) for indicated time points and cell lysates were immunoprecipitated with LARG antibody and subjected to Western blot analysis using DC-SIGN and 4G10 (anti-phosphotyrosine) antibodies. LARG served as a loading control. AbC = negative control; TCL = Total cell lysate. Results are representative of 3 independent experiments. (**f**) Quantitative analysis of the Western blots of DC-SIGN/LARG, DC-SIGN/LSP1, LARG/4G10 and LARG/DC-SIGN interactions. The band intensity in each lane was determined by Adobe Photoshop. The fold change was determined by calculating the value of each lane vs. the unstimulated control (0 h) Data represent the mean ± SD of 3 independent experiments (**p ≤ 0.01, 2-tailed t-test).

The binding of DC-SIGN to HIV-1 gp120 or to a specific antibody or ligand will activate DC-SIGN signaling, primarily mediated by the Rho guanine nucleotide-exchange factor LARG^34^. Activated LARG recruits the scaffold protein Leucocyte Specific Protein (LSP) 1 and forms the signalosome complex which plays a crucial role in internalization of virus particles into intracellular non-lysosomal compartments^27^,^34^. Since we observed increased expression of DC-SIGN and increased internalization of HIV-1 in cocaine treated cells, we studied the effects of cocaine on the DC-SIGN/LARG/LSP1 complex. We pretreated the DCs with cocaine and infected with HIV-1 for 24 hours, and then analyzed DC-SIGN interactions with LARG and LSP1 by confocal microscopy. We observed increased interaction between DC-SIGN and LARG in HIV-1 inoculated cells compared to uninoculated cells (Fig. 4a,c, p = 0.013). This interaction was significantly enhanced in cocaine pretreated and HIV-1 inoculated cells compared to cells inoculated with HIV-1 alone (Fig. 4a,c, p = 0.009). Interestingly, cells treated with cocaine alone also exhibited an increased interaction between DC-SIGN and LARG compared to control cells (UN) (Fig. 4a,c, p = 0.016). Further, we studied the effects of cocaine on DC-SIGN and LSP1 interaction by confocal microscopy. We observed a significant increase in interaction between DC-SIGN and LSP1 in cocaine pretreated HIV-1 incubated cells compared to HIV-1 incubated cells (Fig. 4b,c, p = 0.0011). Interaction between DC-SIGN and LSP1 also was significantly enhanced upon incubation with cocaine alone (p = 0.005) or HIV-1 alone (p = 0.023) (Fig. 4b,c) compared to control cells. These results indicate that cocaine enhances formation of a DC SIGN/LARG/LSP1 complex, which plays a role in HIV-1 internalization.

To further confirm cocaine itself can induce the interaction between DC-SIGN and LARG, we collected cell lysates from cells with or without cocaine treatment at various time points. After DC-SIGN immunoprecipitation, we examined the interaction of LARG and LSP1 to DC-SIGN by Western blot analysis. An increased interaction of DC-SIGN with LARG (p = 0.008) and DC-SIGN and LSP1 (p = 0.0085) was observed in cocaine treated cells compared to control treated cells (Fig. 4d,f). Activity of LARG is determined by its phosphorylation status. Hence we analyzed the phosphorylation status of LARG by Immunoprecipitation and Western blot analysis with phosphotyrosine antibody and revealed that an increased phosphorylation of LARG in cocaine treated DCs compared to untreated cells (Fig. 4e,f, p = 0.002). We also confirmed increased association of DC-SIGN and LARG upon cocaine treatment by LARG immunoprecipitation (Fig. 4e,f, p = 0.001).

### LARG is crucial for HIV-1 internalization in DCs

**Figure 5:**
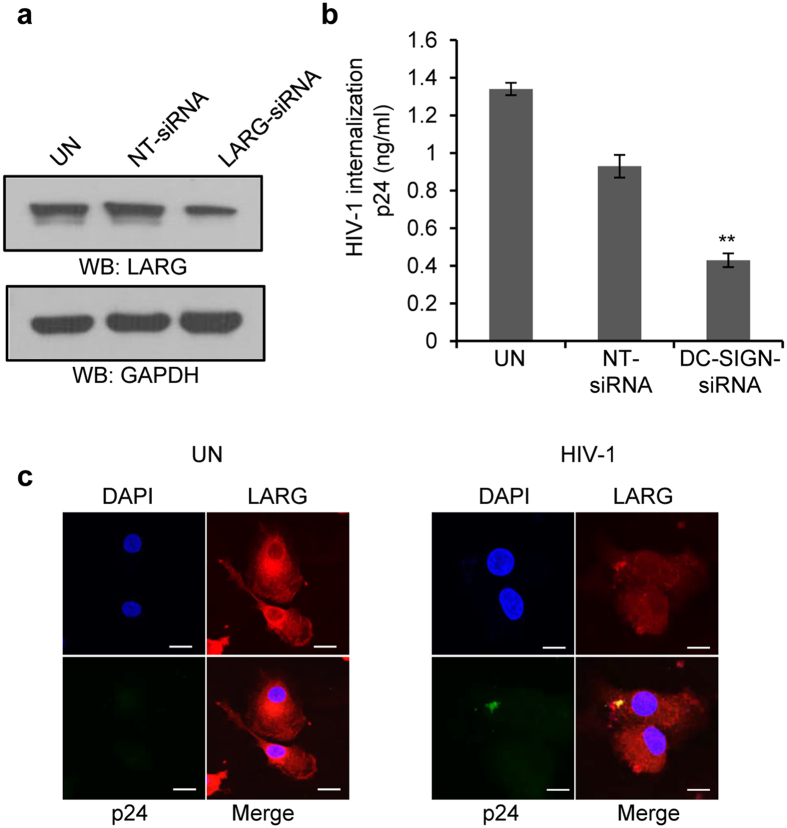
The role of LARG in internalization of HIV-1 in DCs. (**a**) Representative Western blot analysis of LARG expression in untreated DCs and in DCs transfected with NT-siRNAs or LARG-specific siRNAs. GAPDH served as a loading control. (NT-non targeted, control). (**b**) HIV-1 p24 titer of HIV-1 internalization assay in untreated DCs and in DCs transfected with NT-siRNAs or LARG-specific siRNAs. Data represent the mean ± SEM of 3 experiments for NT-siRNA transfected DCs vs. LARG- siRNA transfected DCs (**p ≤ 0.01, 2-tailed t-test). (**c**) Confocal images of LARG (Red) and HIV-1 p24 (Green) localization in HIV-1 infected or uninfected DCs. Results are representative of 3 independent experiments. Scale bars = 10 μm.

DC-SIGN mediated activation of LARG and its recruitment to a DC-SIGN complex appear to be required for viral sequestration and formation of an infectious synapse between DCs and T-cells^34^. We investigated the role of LARG in the internalization of HIV-1 by DCs. To that end, we knocked down LARG expression in DCs by using specific siRNA (Fig. 5a) and then performed an HIV-1 internalization assay. We found that HIV-1 internalization was significantly inhibited in LARG-siRNA transfected cells compared to non-targeted si-RNA transfected cells (Fig. 5b, p = 0.002). Further, we analyzed HIV-1 and LARG localization by confocal microscopy after exposing HIV-1 to DCs for 4 hours. We found co-localization between HIV-1 and LARG (Fig. 5c), indicating that LARG may play a role in DC-SIGN mediated internalization of HIV-1 by DCs.

### Cocaine induces HIV-1 localization to VPS4 and CD81 and inhibits HIV-1 localization to LAMP1

**Figure 6:**
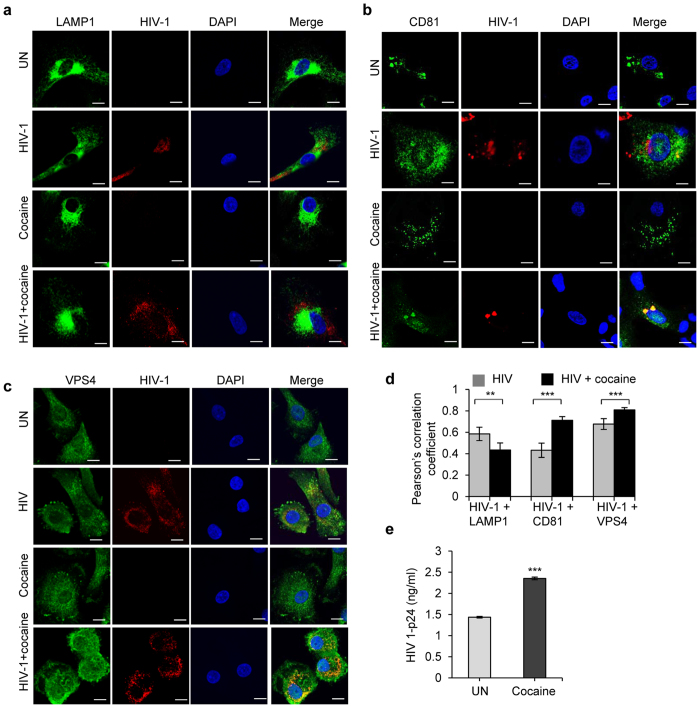
Cocaine enhances HIV-1 co-localization to CD81 and VPS4 and inhibits HIV-1 co-localization to LAMP1. (**a–c**) Confocal images of the co-localization of HIV-1 p24 with LAMP1 (**a**) or CD81 (**b**) or VPS4 (**c**) in untreated DCs and DCs treated with cocaine (1 μM) or incubated with HIV-1 or treated with cocaine and incubated with HIV-1. Images are representative of 3 independent experiments (Scale bars = 10 μM). (**d**) Quantitative analysis of the colocalization of HIV-1 p24 with LAMP1 or CD81 or VPS4, under conditions identical to (**a–c**), using confocal microscopy and Image J2 software. Data represents mean of Pearson’s correlation coefficient indices of 10 randomly chosen images per condition (**p ≤ 0.01, ***p ≤ 0.001, 2-tailed t-test). (**e**) HIV-1 p24 titer of HIV-1 degradation assay as described in Methods in DCs untreated or treated with cocaine for 3 hours. Data represent the mean ± SEM of 3 experiments done in triplicate for untreated DCs vs. cocaine treated DCs (***p ≤ 0.001, 2-tailed t-test).

The fate of internalized HIV-1 varies depending on its intracellular trafficking. Most of the internalized viral particles undergo degradation at phagolysomal complexes, while some avoid degradation and are trafficked into multivesicular bodies; this detour may contribute to viral transmission or integration of viral genome into host genome with further replication^29^,^30^,^31^,^32^,^33^. Several molecules regulate intracellular trafficking of internalized virus^44^. We studied the intracellular trafficking of HIV-1 in the presence and absence of cocaine via its interaction with markers of the intracellular compartments. We analyzed the co-localization of HIV-1 p24 with lysosomal-associated membrane protein-1 (LAMP1) by confocal microscopy and found a significant decrease in colocalization of HIV-1 p24 and LAMP1 in cocaine treated cells compared to untreated cells (Fig. 6a,d, p = 0.01). Further analysis revealed that there was increased co-localization of HIV-1 and CD81, which belongs to group of tetraspanins and is a marker for endosome or multivescicular bodies (MVB) (Fig. 6b,d, p = 0.0003)^31^. We further confirmed endosomal localization of HIV-1 using VPS4 as a marker. VPS4 protein is a member of the AAA protein family (ATPases associated with diverse cellular activities) and a key player in the transport of proteins out of a prevacuolar endosomal compartment^45^,^46^. Confocal analysis revealed increased colocalization of VPS4 and HIV-1 p24, indicating that HIV-1 localizes preferentially on endosomes or MVBs in cocaine treated DCs (Fig. 6c,d, p = 0.00003). These results indicate that cocaine may affect the intracellular trafficking HIV-1 and its degradation. Next, we performed a viral degradation assay to analyze the effect of cocaine on HIV-1 degradation in DCs. We observed a significant increase in intracellular HIV-1 p24 titer after 3 hours in cocaine treated cells compared to control cells. This indicates that cocaine altered the intracellular degradation of HIV-1 in DCs (Fig. 6e, p = 0.00056).

### Cocaine induced activation of Rho A and focal adhesion molecules in DCs and enhanced infectious synapse formation between DCs and T-cells

**Figure 7:**
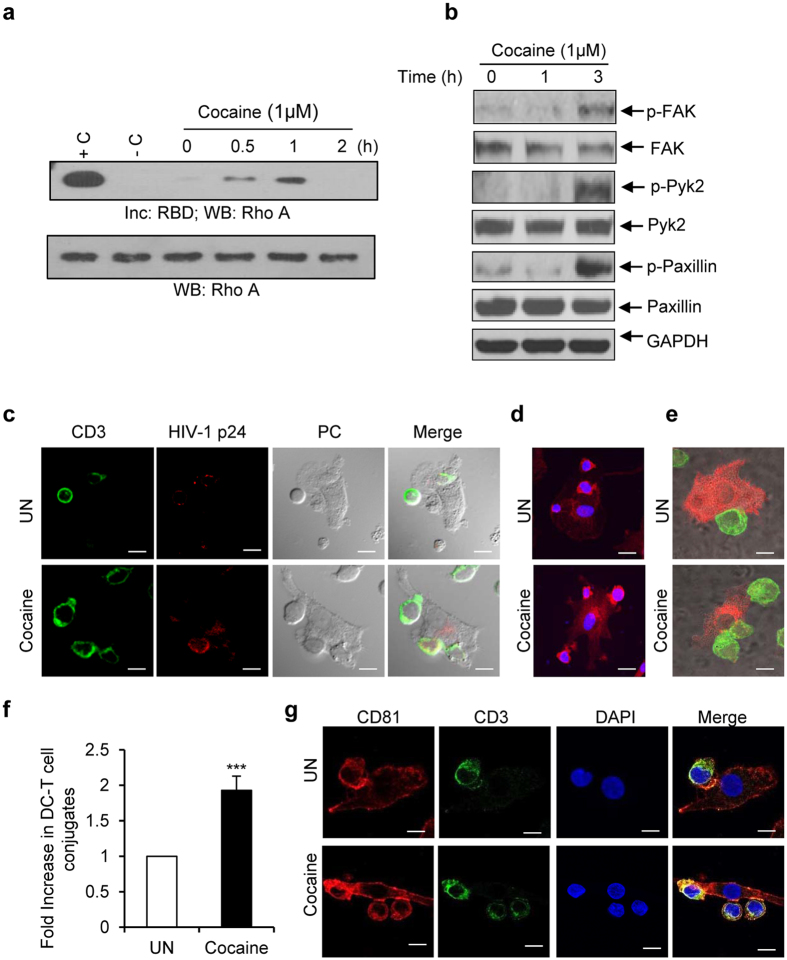
Cocaine activates Rho A, increases phosphorylation of Pyk2, FAK and paxillin and enhances infectious synapse formation. (**a**) Rho A activity assay: Lysates from DCs untreated or treated with cocaine for various time points were incubated with GST-tagged Rhotekin-RBD (Rho binding domain)-agarose beads. Activated Rho A was quantitated by RBD pull-down and Western blot analysis with Rho A antibody and representative image from 3 independent experiments was shown. Total Rho A in total cell lysate used as loading control. (**b**) Representative Western blot analysis of p-FAK, FAK, p-Pyk2, Pyk2, p-paxillin, and paxillin expression in untreated DCs and DCs treated with cocaine (1 μM) for indicated time points. GAPDH used as loading control. (**c**) Representative phase contrast/fluorescent microscopic images of infectious synapse in DCs infected with HIV-1 BaL and co-cultured with autologous T-cells in the absence (upper panel) and in the presence of cocaine (lower panel). (Green = CD3; Red = HIV-1 p24, Scale bars = 10 μm. (**d** and **e**) Representative confocal images of DC and T-cell infectious synapse by membrane staining with Rhodamine phalloidin (**d**) and staining with DC-SIGN (Red) and CD3 (Green) (**e**) in DCs infected with HIV-1 BaL and co-cultured with autologous T-cells in the absence (upper panel) or in the presence of cocaine (lower panel). Scale bars = 10 μm. (**f**) Fold increase in DC and T-cell conjugates in the slides from above mentioned experiments. Total number DC and T-cell conjugates were counted in 25, 63X oil immersion fields and fold increase were calculated by considering untreated as 1. Data indicate the mean ± SD of 3 independent experiments (***p ≤ 0.001, 2-tailed t-test). (**g**) Representative confocal images of infectious synapse by staining with anti- CD81(Red) and anti-CD3 (Green) antibodies in DCs infected with HIV-1 BaL and co-cultured with autologous T-cells in the absence (upper panel) or in the presence of cocaine (lower panel). Scale bars = 10 μm.

Since we observed increased phosphorylation of LARG in cocaine treated DCs, we further investigated regulation of its downstream signaling molecules. Activation of LARG is known to enhance activity of Rho-GTPases, and this can play a role in the formation of an infectious synapse between DCs and T-cells^34^. Hence, we tested the activity of CDC42, Rac1 and Rho A in cocaine treated DCs at various time points. We found that cocaine increased the activity of Rho A (Fig. 7a), but did not observe changes in the activation status of CDC42 and Rac1 upon cocaine treatment (data not shown).

The infectious synapse or immune synapse is composed of various focal adhesion molecules including paxillin, talin and vinculin^47^. Hence, we analyzed the effects of cocaine on phosphorylation of focal adhesion molecules such as FAK, Pyk2 and paxillin in DCs and found that increased phosphorylation of these molecules in cocaine treated cells (Fig. 7b). This suggests cocaine may enhance synapse formation via its effects on paxillin, FAK and Pyk2.

DC-SIGN and its downstream mediators play a key role in infectious synapse formation and cell-cell transmission of HIV-1^34^. We thus studied infectious synapse formation in DCs incubated with HIV-1 BaL, co-culturing autologous T-cells, in the presence or absence of cocaine. We analyzed the immune synapse by using various staining techniques. To identify the HIV-1 mediated DC-T infectious synapse conjugates, we stained CD3 as a T-cell specific marker and HIV-1 p24 and analyzed by phase contrast microscope/fluorescent microscopy to differentiate DC from T-cells by shape and size. Analyses of merged images indicated increased infectious synapse formation in cocaine treated cells compared to control cells (Fig. 7c). Further, we analyzed infectious synapses by staining the cell membrane and staining specifically DCs by DC-SIGN and T-cells by CD3. We observed a significant increase in DC and T-cell conjugates in cocaine treated cells compared to control cells (Fig. 7d–f, p = 0.00084). Tetraspanins play major roles in the trafficking of HIV-1 to the DC and T-cell infectious synapse^29^. Hence we analyzed infectious synapse by staining using CD81 and CD3 antibodies. We found an increase in localization of CD81 at DC and T-cell infectious synapses and further observed cocaine enhanced the localization of CD81 at the junctions of DC and T-cells conjugates (Fig. 7g). These results confirm that cocaine enhances infectious synapse formation between DCs and T-cells and facilitates HIV-1 transfer.

## Discussion

Cocaine is a prominent co-factor in the pathogenesis of HIV-1 infection and the progression to AIDS^1^,^2^,^3^,^4^,^5^,^6^,^7^. Risky behavior associated with cocaine increases the probability of acquiring HIV and undermines adherence to treatment^1^,^2^,^3^,^4^,^5^,^6^,^7^. However, independent of behavioral factors, studies indicate enhanced replication and transmission of HIV-1 among cocaine using HIV-1 infected individuals^4^,^8^,^9^,^10^,^11^,^12^,^13^,^14^,^15^,^16^,^17^,^18^.

We sought to investigate the effects of cocaine on cell-to-cell transmission of HIV-1. We found that cocaine enhanced DC to T-cell transmission of HIV more than 2.5 fold; however we did not observe significant changes in HIV transfer from T-cells to T-cells due to the drug. Moreover, our analysis of HIV-1 supernatants of DC-to-T and T-cell to T-cell cultures revealed a significant increase in HIV-1 p24 titer in both, indicating that cocaine can enhance HIV-1 replication in T-cells. Although cocaine exerted an effect on the immune synapse formed between DC and T-cells, it showed a distinct effect on the viral replication machinery in T-cells. These results suggest that cocaine may modulate different stages of the viral life cycle in different cell types.

Dendritic cells are primary antigen-presenting cells that capture HIV in the peripheral tissues of mucosal surfaces and migrate to the secondary lymphoid organs where they prime T-cells or transmit the infection^39^. DCs sequester HIV-1 virions in multi vesicular bodies through their c-type lectin receptor DC-SIGN^24^,^25^,^26^. In DCs, DC-SIGN can function as an adhesion receptor, fostering DC-T-cell interaction via its binding to intercellular adhesion molecule 3 (ICAM-3)^26^. DC-SIGN also can play a role in the recognition and binding of HIV-1 via the gp120 glycoprotein, resulting in the internalization of virus^24^,^25^,^26^. Furthermore, DC-SIGN mediated capture and internalization of HIV-1 particles by immature DCs alters the intracellular trafficking machinery, evades lysosomal degradation pathways and subsequently transfers virus to T-cells through infectious synapse^28^,^35^,^39^,^48^,^49^,^50^. Several studies demonstrated that blocking of DC-SIGN significantly inhibited HIV-1 transmission from DC to T-cells^51^,^52^. However, studies have also shown that DC-SIGN mediated internalized HIV-1 particles undergo degradation and subsequent presentation of HIV-1 antigen to T-cells^53^,^54^,^55^. Recent research indicates that surface-bound HIV-1 on DCs is more effectively transmitted to T-cells, rather than the internalized HIV-1 particle^31^,^37^,^56^,^57^,^58^.

In our present study, after observing an increase in DC-T transmission of HIV-1, we sought to explore the molecular mechanisms involved in the phenomenon. Both HIV-1 binding and internalization were significantly enhanced by cocaine. We then analyzed effects of the drug on several of the innate host resistance factors in DCs. We did not observe any changes in the expression of APOBEC3G and SAMHD1 upon cocaine treatment, but found decreased expression of tetherin.

Previous studies showed increased DC-SIGN expression in cells derived from cocaine using HIV-1 positive individuals, as well as in DCs derived from cocaine using healthy individuals upon treatment with HIV-1 or HIV-1 gp120 or tat^23^. In our study, we analyzed effects of cocaine alone on DC-SIGN expression in DCs derived from healthy individuals. Consistent with earlier work, we found that cocaine alone can significantly enhance DC-SIGN expression in DCs. In addition, we analyzed HIV-1 co-receptors CXCR4 and CCR5 in DCs and T-cells after cocaine treatment, however we did not observe significant changes in these chemokine receptors except a moderate increase in CXCR4 expression in cocaine treated DCs.

In unstimulated DCs, DC-SIGN is constitutively associated with scaffolding proteins including LSP1, CNK, Raf1and KSR1; these molecules form a ‘signalosome complex’ which plays a pivotal role in the induction of pathogen specific immune responses^27^. Binding of HIV-1 gp120 to DC-SIGN activates DC-SIGN signaling which is mainly mediated by Rho guanine nucleotide-exchange factor LARG followed by activation of Rho-GTPases^34^. In our study, we found that cocaine enhanced HIV-1 mediated association of DC-SIGN and LARG. Notably, we also found that cocaine also can induce activation of LARG by its phosphorylation, and induce its association with DC-SIGN. In addition, our experiments in LARG knockdown DCs revealed that LARG may play a key role in the internalization of HIV-1 by DCs. Furthermore, our analysis of intracellular localization of HIV-1 in cocaine treated cells revealed that the drug induced the increased localization of HIV-1 in endosome compartments. This was demonstrated by co-localization of virus with CD81 and VPS4. Interestingly, cocaine also blocked trafficking of HIV-1 towards a lysosomal complex. Efficient degradation of HIV-1 typically takes place in the mature DC phago-lysosomal complex^29^,^32^,^33^. HIV-1 can also alter the trafficking machinery so that a small portion of internalized virus is transmitted in *trans* via an infectious synapse^29^,^31^,^32^,^33^,^39^,^40^. Our study reveals that cocaine can alter the intracellular trafficking machinery in DC, thereby decreasing viral degradation and enhancing viral localization in endosomal compartments or in multi vesicular bodies, which facilitates enhanced viral transfer to T-cells via an infectious synapse.

Cocaine is known to regulate Rho-GTPases activity in various cell types^59^,^60^. Our examination of downstream signaling molecules of the activated DC-SIGN/LARG complex revealed activation of Rho A, a member of Rho-GTPases as well as other focal adhesion molecules FAK, Pyk2 and paxillin. Activated Rho A is known to play a key role in the formation of infectious synapses^34^. We found that infectious synapse formation was significantly enhanced in cocaine treated cells and hypothesize that cocaine induced activation of Rho A results in phosphorylation of LARG, which leads to its co-localization with DC-SIGN and mediates increased internalization of DC-SIGN bound HIV-1. In addition, activated Rho A may enhance the formation of an infectious synapse between DCs and T-cells resulting in increased transmission.

Our study demonstrates for the first time that cocaine enhances cell-cell transmission of HIV-1 via facilitating an infectious synapse between HIV-1 infected DCs and T-cells. Further we elucidated a novel mechanism by which cocaine may exert its effects on HIV-1 pathogenesis by activation of Rho A which enhances DC-SIGN/LARG mediated internalization of HIV-1, and alters the intracellular trafficking of HIV-1. Each of these molecular and functional changes induced by cocaine work in favor of virus incorporation, survival, and transmission. Charting these alterations in primary target cells of HIV can inform strategies to interrupt the virus life cycle in substance using hosts.

## Methods

### Cells, HIV-1 and constructs

Buffy coats were obtained from the Blood Transfusion Service, Massachusetts General Hospital, Boston, MA, in compliance with the Beth Israel Deaconess Medical Center Committee on Clinical Investigations (CCI) protocol #2008-P-000418/5. Buffy coats were provided at this institution for research purposes; therefore, no informed consent was further needed. In addition, buffy coats were provided without identifiers. This study was approved by Beth Israel Deaconess Medical Center’s CCI, Institutional Review Board, and Privacy Board appointed to review research involving human subjects. The experimental procedures were carried out in strict accordance with approved guidelines.

Human peripheral blood mononuclear cells were isolated from buffy coats and monocytes were isolated using a positive selection kit per manufacturer’s protocol (STEMCELL Technologies, Inc.). Monocyte derived dendritic cells were prepared and cultured as previously described^61^. Autologous T-cells were isolated from human peripheral blood mononuclear cells, activated with PHA-L (1 μg/ml) and maintained in complete culture medium supplemented with IL-2 (30 U/ml) (Advanced Biotechnologies, Inc., Columbia, MD) at 2 × 10^6^ cells/ml. Purity of these T-cells was analyzed using CD3 and CD4 staining and flow cytometry.

HIV-1 BaL^62^ and HIV-1 Gag-iGFP^63^,^64^ were obtained from the NIH AIDS Research and Reference Reagent Program, National Institute of Allergy and Infectious Diseases, NIH. To prepare HIV-1 stocks, PBMC derived T-cells were cultured with HIV-1 BaL for 7 days. Fresh T-cells, suspended at 1 × 10^6^ cells/ml were added at day 7. At day 14 after initial viral inoculation, the supernatant was harvested and stored at −80 °C. p24 viral antigen in the supernatants was quantified by ELISA (Perkin Elmer Life and Analytical Sciences, Shelton, CT).

### Antibodies and reagents

Cocaine hydrochloride was procured from Sigma-Aldrich Co. (St. Louis, MO), DC-SIGN, LAMP1, VPS4 and SAMHD1 antibodies were obtained from Cell Signaling Technology (Danvers, MA). APOBEC3G, CD81, Tetherin and GAPDH antibodies were obtained from Santa Cruz Biotechnology, Inc. (Santa Cruz, CA). LARG and 4G10 antibodies were obtained from EMD Millipore (Billerica, MA), Rhodamine-Phalloidin (R415) and CellTrace Far-Red DDAO-SE (C34553) were obtained from Life Technologies Corp. FITC-conjugated p24 GAG (6604665) antibody was obtained from Beckman Coulter, Inc. (Brea, CA). PerCP/Cy5.5-conjugated CXCR4, FITC-conjugated CCR5 and AlexaFluor 647-conjugated DC-SIGN antibodies were obtained from BioLegend (San Diego, CA). Anti-GFP antibody (FITC) was obtained from Abcam (Cambridge, MA).

### Assessing HIV-1 transfer by flow cytometry and HIV-1 p24 ELISA

To analyze the effect of cocaine on HIV-1 transfer from DCs to T-cells and T-cell to T-cells, cocaine pre-treated (1 μM for 2 hours) DCs (2.5 × 10^5^) or T-cells (1.0 × 10^6^) were incubated with HIV-1 BaL [20 ng/ml p24 gag] for 2 hours, washed thrice in 1X PBS to remove untrapped virions, and replated. Subsequently, Far-Red-labeled T-cells (1.0 × 10^6^) were added, with and without cocaine. After indicated times, cells were stained for p24-FITC (HIV-1 marker) before acquiring by flow cytometry. Far-Red-labeled T-cells were analyzed for p24 co-expression by FACS analysis, from a total of 5,000 acquired events. Cell supernatants were collected at indicated time points and HIV-1 p24 titer was quantitated by using ELISA (Perkin Elmer, Waltham, MA). HIV-1 Gag-iGFP virus transfer assay was performed by co-culturing the HIV-1 Gag-iGFP nucleofected Jurkat T-cells with Far-Red-labeled Jurkat T-cells (Jurkat T-cells were obtained from American Type Culture Collection, Manassas, VA) in the presence or absence of cocaine (1 μM). After 3 days, Far-Red-labeled T-cells were analyzed for GFP co-expression by FACS analysis, from a total of 10,000 acquired events.

### Western blotting and immunoprecipitation

iMDDCs were left untreated, or treated with cocaine in indicated concentration. After indicated incubation time, samples were collected in RIPA buffer. Protein lysates were separated on NuPAGE precast gels (Life Technologies Corp.), transferred to 0.45 μm nitrocellulose membranes (Bio-Rad Laboratories, Hercules, CA), and probed with appropriate primary antibodies followed by incubation with their respective secondary antibodies. Proteins were visualized with Western Lightning Plus ECL Substrate (PerkinElmer, Waltham, MA).

For immunoprecipitation assay, DCs were left untreated or treated with cocaine (1 μM) and incubated for times indicated. DCs were lysed with cell lysis buffer (Cell Signaling Technology). Immunoprecipitation was performed as previously described^61^.

### Virus binding and internalization assay

The assay was performed as described previously to detect the binding and internalization of HIV-1 particles into MDDCs^65^. Briefly, DCs (1.0 × 10^6^ cells/ml) were untreated or pre-treated with cocaine (1 μM) at 37 °C for 2 hours then incubated with HIV-1 BaL (20 ng/ml, p24) for 2 hours at 4 °C, and unbound virus particles were removed by washing the cells three times in 1X PBS. DCs were lysed with cell lysis buffer (RIPA containing 1% Triton X-100). Viral binding was estimated by quantitating the p24 in the cell lysates by ELISA. Total cell protein was estimated and all samples were normalized for protein content.

For internalization assay, DCs (1.0 × 10^6^ cells/ml) were incubated with HIV-1 (20 ng/ml, p24) in the presence or absence of cocaine (1 μM) for 4 hours at 37 °C, and unbound virus particles were removed by washing the cells three times in 1X PBS. The cells then incubated with 0.05% trypsin for 5 minutes to remove surface bound viral particles. DCs were lysed with cell lysis buffer (RIPA containing 1% Triton X-100). Viral binding was estimated by quantitating the p24 in the cell lysates by ELISA. Total cell protein was estimated and all samples were normalized for protein content.

### Viral degradation assay

The assay was performed as described previously^66^. Briefly, DCs (1.0 × 10^6^ cells/ml) were incubated with HIV-1 (20 ng/ml, p24) in the presence or absence of cocaine (1 μM) at 37 °C. Following 2 hours of incubation, cells were washed and treated with 0.05% trypsin for 5 minutes to remove surface bound viral particles, then incubated at 37 °C with or without cocaine for 3 hours. Cell lysis was performed and the intracellular p24 content was measured by using ELISA.

### siRNA-Mediated Knockdown of LARG

Small interference RNA-mediated knockdown of LARG was performed using LARG –siRNA (Santa Cruz Biotechnology). A non-targeting siRNA (Qiagen, Valencia, CA) was used as the negative control. siRNAs were transfected into iMDDCs by nucleofection (Lonza Wakersville Inc, Wakersville, MD) per manufacturer’s instructions.

### CDC42 and Rac1 Activation Assay

CDC42 and Rac1 activation were determined using the Rac/Cdc42 activation assay kit (SGT445, Upstate Biotechnology, Waltham, MA). In brief, cell lysates were incubated with 15 μg/ml p21-activated kinase (PAK)-1 agarose for 1 hour at 4 °C, per manufacturer’s instructions. Agarose beads were collected by centrifugation, followed by denaturation, boiling of the samples, and SDS-PAGE analysis. Proteins were transferred to nitrocellulose membranes, and Western blot analysis was performed using mouse anti-human CDC42 or Rac1 antibody.

### Confocal Microscopy and Infectious Synapse Assay

DCs were cultured on chamber slides. They were untreated or pretreated with cocaine (1 μM) for 2 hours, and then incubated with HIV-1 BaL for 24 hours. They were fixed in 4% paraformaldehyde and blocked with 5% normal goat serum in PBS/Triton X100 (1 hour). Cells were then incubated with primary antibodies overnight at 4 °C, washed thrice with PBS, and stained with AlexaFluor 568–labeled anti–mouse IgG antibody (Molecular Probes®; Invitrogen) and/or Rhodamine-phalloidin (Molecular Probes) for 2 hours. Subsequently, cells were washed thrice with PBS, and slides were mounted using Prolong Gold antifade with DAPI (4′,6-diamidino-2-phenylindole; Invitrogen). Slides were examined under a Zeiss 880 Meta confocal microscope (Carl Zeiss Microimaging, LLC, Thornwood, NY), and images were acquired using ZEN2 software (Carl Zeiss). Figures were made using Adobe Photoshop CS4 software (Adobe Systems, San Jose, CA).

Quantification of colocalization: Colocalization analysis of the different proteins was performed using ImageJ2 software and the Coloc2 plugin, which measures Pearson’s Correlation Coefficient (PCC) based on the pixel intensity of the red and green probes. Ten individual cells were randomly chosen for each condition and regions of interest were outlined over the images; colocalization was quantitated by measuring PCC.

For Infectious synapse assay: DCs were cultured and pretreated with cocaine as mentioned above, and then incubated with HIV-1 BaL for 2 hours. Cells were washed and autologous T-cells were added in 1:4 ratio. After 24 hours of incubation at 37 °C, cells were fixed, stained and examined under confocal microscope as mentioned above. DC-T-cell conjugate formation was quantified by counting DC-T-cell conjugates in 25 fields.

### Statistics

Differences between groups were calculated using a standard 2-tailed Student’s t-test. p-values ≤ 0.05 were considered statistically significant.

## Additional Information

**How to cite this article**: Prasad, A. *et al*. Cocaine Enhances DC to T-cell HIV-1 Transmission by Activating DC-SIGN/LARG/LSP1 Complex and Facilitating Infectious Synapse Formation. *Sci. Rep.* **7**, 40648; doi: 10.1038/srep40648 (2017).

**Publisher's note:** Springer Nature remains neutral with regard to jurisdictional claims in published maps and institutional affiliations.
